# Perioperative use of gabapentinoids for the management of postoperative acute pain: protocol of a systematic review and meta-analysis

**DOI:** 10.1186/s13643-018-0906-3

**Published:** 2019-01-16

**Authors:** Michael Verret, François Lauzier, Ryan Zarychanski, Xavier Savard, Marie-Joëlle Cossi, Anne-Marie Pinard, Guillaume Leblanc, Alexis F. Turgeon

**Affiliations:** 1 Department of Anesthesiology and Critical Care Medicine, Division of Critical Care Medicine, Université Laval, Québec, QC Canada; 2 CHU de Québec - Université Laval Research Center, 1401, 18e rue, Québec, Québec G1J 1Z4 Canada; 3 Department of Medicine, Université Laval and CHU de Québec - Université Laval Research Center, Québec, QC Canada; 4 Cancer Care Manitoba, Department of Hematology and Medical Oncology, Winnipeg, MN Canada; 5 CHU de Québec - Université Laval (Hôpital de l’Enfant-Jésus), 1401, 18e rue, Québec, Québec G1J 1Z4 Canada

**Keywords:** Gabapentinoids, Acute pain, Analgesia, Postoperative, Surgery, Anesthesiology

## Abstract

**Background:**

Opioids are commonly used for the management of postoperative pain, but their use is limited by important adverse events, such as respiratory depression and the potential for addiction. Multimodal opioid-sparing analgesia regimens can be effectively employed to manage postoperative pain and reduce exposure to opioids. Gabapentinoids (pregabalin and gabapentin) represent an attractive class of drugs for use in multimodal regimens. The American Pain Society recommends the use of gabapentinoids during the perioperative period; however, evidence to inform such a recommendation is unclear.

**Methods:**

We will conduct a systematic review and meta-analysis of randomized clinical trials evaluating the use of systemic gabapentinoids, in comparison to other analgesic regimens or placebo in adult patients undergoing surgery. We will search MEDLINE, Embase, the Cochrane Central Register of Controlled Trials (CENTRAL), the Web of Science, and ClinicalTrials.gov databases for relevant citations. Our primary outcome will be intensity of postoperative acute pain (12 h). Our secondary outcomes will be postoperative pain intensity at 6, 24, 48 h, and 72 h, cumulative dose of opioids administered within 24, 48, and 72 h following surgery, the length of stay, chronic pain, and adverse events. Two investigators will independently select trials and extract data. We will evaluate the risk of bias of included trials using the Cochrane risk of bias tools. We will represent pooled continuous data as weighted mean differences and pooled dichotomous data as risk ratios with a 95% confidence interval. We will use random effect models and assess statistical heterogeneity with the *I*^2^ index.

**Discussion:**

Our study will provide the best level of evidence to inform the effect of gabapentinoids in the management of postoperative acute pain.

**Systematic review registration:**

PROSPERO CRD42017067029

**Electronic supplementary material:**

The online version of this article (10.1186/s13643-018-0906-3) contains supplementary material, which is available to authorized users.

## Background

Because of their numerous adverse events (nausea and vomiting, respiratory depression, pruritus, sedation, etc.) and their potential for addiction, reducing opioid administration is a core element in the domain of postoperative pain management [1–3]. In this context, multimodal analgesia (opioid sparing analgesia) is widely used in perioperative medicine in order to reduce opioid use while still providing optimal postoperative pain management. In the last decade, gabapentinoids (pregabalin and gabapentin) emerged as an increasingly used alternative for the management of postoperative acute pain, despite the lack of approval by health authorities for this indication [4–8].

The American Pain Society supports the use of gabapentinoids as a part of multimodal analgesia [9]. However, the literature does not support this recommendation. In a systematic review published in 2007 that included 22 trials, representing 1909 patients, gabapentinoids had a statistically significant opioid sparing effect (WMD, -30 mg of oral morphine equivalent; 95% CI, -26 to -34) [10]. Interpretation of the results was limited by the small number of trials and substantial heterogeneity despite subgroup analyses. Since this study, systematic reviews were carried out only on a specific type of drug (pregabalin or gabapentin) or a specific type of surgery [11–24]. These systematic reviews observed a mitigated benefit from the use of gabapentinoids and raised concerns about serious adverse events such as respiratory depression and sedation [11, 15, 25, 26]. To date, there is no recent systematic review evaluating the efficacy and safety of gabapentinoids in different surgical settings. In this context, a high quality and updated systematic review on this class of drug is warranted to inform accurate recommendations for its perioperative utilization.

## Methods

### Aims

Our primary objective is to evaluate the analgesic effect of gabapentinoids in perioperative care in adult surgical patients compared to placebo or any other analgesic regimen. Our secondary objectives are to assess its effect on the opioid use, the length of stay and the incidence of chronic pain. We will also evaluate the incidence of adverse events.

### Study design

Our study is a systematic review and meta-analysis of randomized controlled trials and our protocol was prepared according to the methodological recommendations of the Cochrane Handbook for Systematic Reviews and Meta analyses [27]. This protocol is registered in PROSPERO CRD42017067029 (http://www.crd.york.ac.uk/PROSPERO/display_record.php?ID=CRD42017067029). The manuscript reporting the results of the systematic review will be written following the Preferred Reporting Items for Systematic Reviews and Meta-Analyses (PRISMA-P2015) recommendations [28].

### Outcome measures

Our primary outcome measure is postoperative pain intensity 12 h after surgery or at the time of hospital discharge if earlier. We decided to prioritize dynamic (during movement) pain over rest pain if both are reported, because this type of pain is more relevant for patients and it is associated with better recovery [29]. We selected the 12-h time frame for postoperative pain assessment because of its clinical relevance and acceptance in the field of acute postoperative pain research [30].

Our secondary outcome measures are the postoperative pain intensity at different time points (6, 24, 48, and 72 h), the cumulative dosage of intravenous (IV) morphine equivalent (ME) over the first 24, 48, and 72 h following surgery, and the lengths of stay (hospital, post anesthesia care unit, intensive care unit, and day care unit). For the measure of pain intensity, if data at the specific time point are unavailable, the following intervals will be considered: 0–6 h, 7–12 h (primary outcome), 13–24 h, 25–48 h, and 49–72 h. We will also evaluate the incidence of postoperative chronic pain (defined as lasting for 3 months or more). In case the incidence of chronic pain is not reported but the incidence of complex regional pain syndrome (CRPS) is, this data will be used. The incidence of postoperative adverse events such as dizziness, fall or ataxia, delirium, addiction or abuse, visual disturbance, respiratory failure (respiratory insufficiency or respiratory depression), and nausea and/or vomiting (PONV) will be considered.

### Eligibility criteria and study selection

We will include trials comparing the systemic use of gabapentinoids (pregabalin and/or gabapentin) to a placebo or any other type of drug used for the management of perioperative acute pain. Our study population will be adult patients (more than 80% of patients are 18 years of age or older) undergoing elective or emergent surgery under any type of anesthesia. To be included, trials should have studied the perioperative use of gabapentinoids (defined as being started at any moment between 1 week prior to and 12 h after surgery) and have evaluated at least one of our outcomes of interest. We will exclude trials comparing the use of gabapentinoids to regional analgesia (peripheral and/or neuraxial). Cross-over trials and trials including patients already receiving gabapentinoids for a chronic pain condition will not be considered for inclusion. We will use no language restriction.

### Information sources and search strategy

We will search MEDLINE, Embase, the Cochrane Central Register of Controlled Trials (CENTRAL), Web of Science, and ClinicalTrials.gov databases for randomized control trials published to date. We will generate Medical Subject Headings (MeSH), Emtree terms, and free text words to identify articles. We will divide vocabulary themes into three main text term categories: (1) perioperative (surgery or anesthesiology), (2) gabapentinoids (pregabalin and/or gabapentin), and (3) RCTs. The search strategy will be reviewed by an information specialist, and we will validate its quality according to PRESS 2015 guideline [31]. Bibliographies of included trials will be reviewed to retrieve pertinent publications. An example of our search strategy is available in the Additional file 1. Two members of the research team (MV and XS) will independently assess trials for eligibility.

### Data abstraction

We will create a standardized data abstraction form with Microsoft Excel and carry out a pilot test on the first ten trials. Two reviewers (MV and XS) will then independently extract data. A third reviewer (AFT) will be consulted in cases of disagreements, and all reviewers will be involved in reaching a common consensus. We will contact the authors when relevant information is missing.

We will extract the following study characteristics: years of publication, countries, number of centers participating (multicenter vs single center), total number of patients randomized, and the number of patients analyzed in each group. As for the patient and surgery characteristics, we will extract the following: age, sex, prior chronic use of opioids and dependence, preoperative pain, type of surgery and anesthesia, type of population (geriatric or not), follow-up period, and surgery setting (ambulatory vs in-hospital). As for the intervention and comparator, we will extract the type, timing, and dose of the first gabapentinoid and comparator intake and the daily dosage, including the duration of treatment. For co-analgesia, we will include information about drugs, routes of administration, and regimen (regular vs. on request). Information concerning outcomes, such as postoperative acute (rest or dynamic) and chronic pain, cumulative ME administration, and length of stay in different hospital units (e.g., hospital, post-anesthesia care unit, intensive care unit, and day care unit), will be collected. Reported adverse events will be extracted, including dizziness, fall or ataxia, delirium, addiction or abuse, visual disturbance, respiratory failure, and PONV. In addition, we will extract information concerning study duration, conflict of interest, funding sources from pharmaceutical industry, and risk of bias items. When results are only presented on a diagram or a graph, data will be extracted using a web application (WebPlotDigitizer) [32].

### Data synthesis

For every time point, pain measurement scores and standard deviation will be converted to a 100-point scale [33]. Outcomes reported with a median and range will be converted into mean ± standard deviation according to a standardized equation [34]. Opioid administration will be calculated by converting the mean ± standard deviation for the opioid used in each study to OME (oral morphine equivalent). Conversion factors are based on a recent international review [35]. We will then calculate the IV ME since this is the most common route of administration in the postoperative period.

### Risk of bias assessment

Two independent reviewers (MV and XS) will evaluate the methodological quality of included trials using the Cochrane Collaboration’s tool for assessing risk of bias [36]. This tool comprises seven domains representing potential sources of bias that the reviewers will rank accordingly: high, low, or unclear risk of bias. For each study, we will report its overall methodological quality using the worst score obtained across the seven domains. We will report the risk of bias in a graphic including all trials included in our meta-analysis. We will use GRADE to report the quality of the evidence of summary estimates of our outcomes [37].

### Statistical analysis

Data will be analyzed with Review Manager; version 5.3.5 (RevMan, The Cochrane Collaboration, Oxford, United Kingdom) using random effects models (Mantel-Haenszel for binary outcomes and inverse variance for continuous outcomes). We will represent pooled continuous data as weighted mean differences (WMD) with a 95% confidence interval and pooled dichotomous data as risk ratios (or odds ratio (OR) in the case of rare events if deemed appropriate). We will perform subgroup and sensitivity analyses to evaluate the robustness of our findings and potential sources of heterogeneity. We hypothesize that the following factors may explain heterogeneity: type of funding (pharmaceutical industry or not), type of surgery (overall type and surgery associated with a greater risk of chronic pain), type of follow-up (inpatient or ambulatory surgery), type of population (previous diagnostic of chronic pain condition, addiction to opioids or not, women or others, and geriatric patients or not), type of anesthesia (general, regional, or others), type of drug (gabapentin or pregabalin or both), the dosage regimen (high dose (pregabalin ≥ 300 mg/day and gabapentin ≥ 900 mg/day), low dose (pregabalin < 300 mg/day and gabapentin < 900 mg/day), or both and single or multiple intake), timing of the intervention (preoperative, postoperative, or both), context of pain assessment (rest, dynamic, or unknown), type of comparator (with an analgesic effect or not), type of co-analgesia (regional analgesia or not, opioids or not, and any co-analgesia or not), and the overall risk of bias (low, high, or unclear) [38, 39]. We will perform all subgroup analyses for our primary outcome. For our secondary outcomes, only type of funding, type of drug, the dosage regimen, the association with an opioid analgesic, and the risk of bias will be carried out. We will assess statistical heterogeneity with the *I*^2^ index [40]. We will consider an *I*^2^ greater than 50% indicative of significant heterogeneity. If deemed appropriate, we will conduct a meta-regression to analyze the effect of the cumulative dosage of gabapentinoids until 12 h after surgery using *R software* [41]. We will explore the potential presence of publication bias using funnel plots for outcomes reported in more than ten trials. We will also perform a trial sequential analysis to evaluate our primary outcome in order to account for random errors due to sparse data and repeated testing (error alpha 5%; beta 20%) [42]. To facilitate the clinical interpretation of our primary outcome, we will then calculate the probability of observing an analgesic effect greater than the minimally important difference, defined as 10 points on a 100-point scale, following the OMERACT recommendations [33]. If appropriate, we will pool results using the inverse variance method and we will calculate relative and absolute effect measures using the *R software* [41, 43]. We will perform sensitivity analyses of this analysis with the thresholds of minimally important difference of 20, 30, and 50 points [33].

## Discussion

Gabapentinoids are widely used as an off-label treatment for the relief of chronic pain condition and are increasingly used for the management of postoperative acute pain, while the level of evidence supporting such usage is unclear [4]. Our systematic review is designed to evaluate the use of a class of drugs on clinically significant outcomes that should be driving practices in perioperative acute pain management. In addition to the effect of the gabapentinoids on pain control, we will also look at whether its use could be associated with adverse events and potential harm. We anticipate observing substantial statistical heterogeneity in our meta-analyses that will be explored with planned subgroup analyses.

Our study will provide an accurate synthesis of the level of evidence for the perioperative use of gabapentinoids.

## Additional file

Additional file 1: Search strategy for MEDLINE/Ovid. (DOCX 85 kb)

## Abbreviations

CRPS: Complex regional pain syndrome
GRADE: Grading of Recommendations, Assessment, Development and Evaluation
IV: Intravenous
LOS: Length of stay
OME: Oral morphine equivalent
OMERACT: Outcomes Measures in Rheumatology
OR: Odds ratio
PRESS: Peer Review of Electronic Search Strategies
PRISMA: Preferred Reporting Items for Systematic Reviews and Meta-Analyses
RCTs: Randomized control trials
WMD: Weighted mean differences
