# Supplementary material for: Perioperative use of gabapentinoids for the management of postoperative acute pain: protocol of a systematic review and meta-analysis
Source: Syst Rev. 2019 Jan 16;8:24. doi: 10.1186/s13643-018-0906-3 (PMC6334388; doi:10.1186/s13643-018-0906-3)
Supplement: Supplementary file 1 — Search strategy for MEDLINE/Ovid. (DOCX 85 kb) [file 13643_2018_906_MOESM1_ESM.docx]

Additional file 1: Search strategy for MEDLINE/Ovid

1. Surgery.fs. OR surgery.ti,ab. OR Surgical.ti,ab.
2. Exp Operating Rooms/ OR Operating.ti,ab. OR operative.ti,ab. OR operation*.ti,ab. OR procedure*.ti,ab.
3. Exp Surgical Procedures, Operative/ OR exp Specialties, Surgical/
4. Exp Perioperative Period/ OR exp Perioperative Care/ OR Perioperative.ti,ab. OR Intraoperative.ti,ab. OR Postoperative.ti,ab. OR Preoperative.ti,ab. OR Peri operative.ti,ab. OR intra operative.ti,ab. OR Post operative.ti,ab. OR Pre operative.ti,ab.
5. Exp Postoperative Complications/
6. Exp Anesthesiology/ OR anesthesiology.ti,ab. OR anaesthesiology.ti,ab. OR anesthesiological.ti,ab. OR anaesthesiological.ti,ab. OR anesthesia.ti,ab. OR anaesthesia.ti,ab.
7. Exp "Anesthesia and Analgesia"/ OR exp Anesthesia Department, Hospital/
8. Exp Anesthetics/ OR anesthetic*.ti,ab. OR anaesthetic*.ti,ab.
9. Exp Adjuvants, Anesthesia/ OR premedication.ti,ab. OR pre medication.ti,ab. OR pre medications.ti,ab.
10. recovery room.ti,ab. OR recovery rooms.ti,ab. OR PACU.ti,ab.
11. exp Postanesthesia Nursing/
12. postanesthesia.ti,ab. OR postanaesthesia.ti,ab. OR preanesthesia.ti,ab. OR preanaesthesia.ti,ab. OR perianesthesia.ti,ab. OR perianaesthesia.ti,ab. OR post anesthesia.ti,ab. OR post anaesthesia.ti,ab. OR pre anesthesia.ti,ab. OR pre anaesthesia.ti,ab. OR peri anesthesia.ti,ab. OR peri anaesthesia.ti,ab.
13. Gabapentin.nm OR Gabapentin.ti,ab. OR Neurontin.ti,ab. OR Convalis.ti,ab. OR Nupentin.ti,ab.
14. "1-(((alpha-isobutanoyloxyethoxy)carbonyl)aminomethyl)-1-cyclohexaneacetic acid".nm. OR "1 (aminomethyl) cyclohexaneacetic acid".ti,ab.
15. exp Pregabalin/ OR Pregabalin.ti,ab. OR lyrica.ti,ab. OR "3-(aminomethyl)-5-methylhexanoic acid".ti,ab.
16. Gabapentinoid*.ti,ab.
17. randomized controlled trial.pt. OR controlled clinical trial.pt. OR randomized.ab. OR placebo.ab. OR drug therapy.fs. OR randomly.ab. OR trial.ab. OR groups.ab.
18. exp animals/ NOT humans.sh.
19. #17 NOT #18

(#1 OR #2 OR #3 OR #4 OR #5 OR #6 OR #7 OR #8 OR #9 OR #10 OR #11 OR #12) and (#13 OR #14 OR #15 OR #16 ) AND #19
